# Rare Malignant Indications for Liver Transplantation: A Collaborative Transplant Study Report

**DOI:** 10.3389/fsurg.2021.678392

**Published:** 2021-12-03

**Authors:** Philipp Houben, Simon Schimmack, Christian Unterrainer, Bernd Döhler, Arianeb Mehrabi, Caner Süsal

**Affiliations:** ^1^Department of General, Visceral, and Transplant Surgery, Heidelberg University Hospital, Heidelberg, Germany; ^2^Institute of Immunology, Heidelberg University Hospital, Heidelberg, Germany; ^3^Transplant Immunology Research Center of Excellence, Koç Üniversitesi, Istanbul, Turkey

**Keywords:** liver transplantation, transplant oncology, hepatocellular carcinoma, cholangiocellular carcinoma, neuroendocrine tumor, liver sarcoma liver transplantation for rare malignancies

## Abstract

**Introduction:** Hepatocellular carcinoma (HCC) is by far the leading malignant indication for liver transplantation (LT). Few other malignancies, including cholangiocellular carcinoma (CCC), metastases from neuroendocrine tumors (NET), and sarcomas of the liver (LSAR), also are commonly accepted indications for LT. However, there is limited information on their outcome after LT.

**Methods:** Graft and patient survival in 14,623 LTs performed in patients with hepatocellular carcinoma, CCC, NET, and LSAR from 1988 to 2017 and reported to the Collaborative Transplant Study were analyzed.

**Results:** The study group consisted of 13,862 patients who had HCC (94.8%), 498 (3.4%) who had CCC, 100 (0.7%) who had NET, and 163 (1.1%) who had LSAR. CCC patients showed a 5-year graft survival rate of 32.1%, strikingly lower than the 63.2% rate in HCC, 51.6% rate in NET, and 64.5% rate in LSAR patients (*P* < 0.001 for all vs. CCC). Multivariable Cox regression analysis revealed a significantly higher risk of graft loss and death due to cancer during the first five post-transplant years in CCC vs. HCC patients (HR 1.77 and 2.56; *P* < 0.001 for both). The same risks were increased also in NET and LSAR patients but did not reach statistical significance.

**Conclusion:** Among patients with rare malignant indications for LT, CCC patients showed significantly impaired graft as well as patient survival compared to HCC patients. The observed differences might challenge traditional decision-making processes for LT indication and palliative treatment in specific hepatic malignancies.

## Introduction

Liver transplantation is the treatment of choice for various pathologic conditions, including acute liver failure, chronic liver insufficiency in cirrhosis, and numerous metabolic diseases. Due to disease progression under immunosuppression, active malignancy is usually a contraindication for solid organ transplantation. Liver transplantation (LT) for malignancies occurring from hepatobiliary cells, such as hepatocellular carcinoma (HCC), is a commonplace practice because the malignant tumor can be resected radically as a complete hepatectomy (1). Since the results of LT in selected HCC with limited tumor burden were demonstrated to be comparable to other benign indications, HCC became one of the main indications for liver transplantation (2).

This has encouraged transplant surgeons and oncologists to extend the indication for LT to other primary or secondary malignancies of the liver. However, the numbers remained generally low or even anecdotal for certain tumors (3). Primary malignancies of the liver, other than HCC and cholangiocellular carcinoma (CCC), are relatively rare. Therefore, the number of potential candidates for LT might be too low to identify these patients as recipients of a liver transplant and to implement specialized programs for the successful treatment of these cases. Additionally, tumors, such as intrahepatic CCC and gall bladder carcinomas, are known to show a different and rather aggressive biological course than HCC with early occurrence of local as well as distant metastases (4–6). In the light of donor organ scarcity, an unacceptably high tumor recurrence rate in CCC has historically been the main limitation for LT (7, 8). The Mayo group achieved excellent results for LT in hilar CCC by applying both strict selection protocols to cases with limited disease burden and a distinct neoadjuvant radio-chemotherapy protocol that included staging operations to rule out lymphatic metastases (9). Even though these results were difficult to reproduce at many other centers, early-stage, non-resectable perihilar CCC has become an accepted indication for LT (10). Based on the achievements noted in HCC and CCC, the concept of “transplant oncology” has been promoted as a multidisciplinary approach for improving patient outcomes by application of transplantation-based surgical methods, cell-based anti-tumor therapy, and a better understanding of the mechanisms of self- and non-self-recognition (11).

Since further development of oncologic strategies in transplantation necessitates a profound understanding of the current state of practice and associated results, we analyzed the outcomes of LT in “non-HCC malignancy” cases from the Collaborative Transplant Study (CTS). The CTS has been collecting data prospectively from more than seven hundred thousand solid organ transplant cases worldwide since 1982. A dedicated follow-up concept and the integration of data obtained from the most important transplant registries guarantee high-quality data. Participation in the CTS is voluntary and is based on cooperative scientific exchange. More than 400 transplant centers from 42 countries contributed to the CTS database. Respect is paid to the confidentiality of recipients and transplant centers alike. Furthermore, the study is a forum to develop interdisciplinary research ideas that are coordinated from the study coordination site at the Institute of Immunology of University Hospital Heidelberg (12).

## Methods

This study examined CTS data from patients who had received a first deceased donor liver transplant between January 1, 1988, and December 31, 2017. Analyses were restricted to patients for whom data regarding recipient age, sex, and original disease were available. Recipients of multi-organ transplants were excluded. The CTS questionnaires contain information on pre-transplant malignancies and a maximum of two different original diseases. Based on these sources of information, the patients were categorized into four groups: HCC, CCC, neuroendocrine tumor (NET), and sarcomas of the liver (LSAR), as reasons for transplantation. The information on the original disease was listed as LSAR for one patient with a pre-transplant NET; therefore, this patient was categorized as having LSAR as the original disease. There was one patient with a pre-transplant CCC who also had CCC and metastasis listed as the original diseases. Thus, this patient was placed in the CCC group. Forty-seven other unclear combinations, such as listings with HCC/CCC or NET/HCC combinations as the original disease, were excluded from the analysis. The demographics of patients are shown in Table 1. CCC, NET, and LSAR patients were younger, more likely to be women, and tended to receive organs from younger donors than HCC recipients. The information on the immunosuppressive protocol was missing in 32.9% of the cases. Calcineurin inhibitor-based immunosuppression was used in 94% of the patients in whom the information was available. The CTS research has been approved by the local Ethics Committee at Heidelberg University (No. 083/2005) and performed in accordance with the principles of the Declaration of Helsinki. General Data Protection Regulation rules of the European Community were adopted in 2018.

**Table 1:** Demographics of the study population.

	**Unknown (%)**	**Original disease malignancy type**					***P***
		**Total**	**HCC**	**CCC**	**NET**	**LSAR**	
		**(*n* = 14,623)**	**(*n* = 13,862)**	**(*n* = 498)**	**(*n* = 100)**	**(*n* = 163)**	
Transplant year	–						<0.001
1988–1997		1,844 (12.6%)	1,565 (11.3%)	217 (43.6%)	22 (22.0%)	40 (24.5%)	
1998–2007		5,650 (38.6%)	5,446 (39.3%)	142 (28.5%)	29 (29.0%)	33 (20.2%)	
2008–2017		7,129 (48.8%)	6,851 (49.4%)	139 (27.9%)	49 (49.0%)	90 (55.2%)	
Recipient age	–						<0.001
Mean (SD)		54.7 (11.2)	55.2 (10.6)	49.3 (11.7)	45.4 (11.6)	28.3 (21.2)	
Recipient sex	–						<0.001
Female		2,812 (19.2%)	2,488 (17.9%)	176 (35.3%)	53 (53.0%)	95 (58.3%)	
Male		11,811 (80.8%)	11,374 (82.1%)	322 (64.7%)	47 (47.0%)	68 (41.7%)	
Donor age	1.9						<0.001
Mean (SD)		50.5 (18.9)	50.9 (18.8)	42.6 (17.7)	46.6 (18.7)	35.6 (17.9)	
Donor sex	1.4						0.006
Female		5,959 (41.5%)	5,636 (41.4%)	201 (42.0%)	46 (46.0%)	76 (48.1%)	
Male		8,388 (58.5%)	7,974 (58.6%)	278 (58.0%)	54 (54.0%)	82 (51.9%)	
Immunosuppressive medication	32.9						<0.001
CNI-based		9,213(94.0%)	8,739 (93.9%)	319 (92.2%)	70(90.9%)	85(95.5%)	
Other		590 (6.0%)	572 (6.1%)	27 (7.8%)	7 (9.1%)	4 (4.5%)	
Steroids							<0.001
Yes		7,568 (51.9%)	7,120 (76.5%)	303 (92.9%)	70 (90.9%)	75 (84.3%)	
No		2,191 (48.1%)	2,191 (23.5%)	23 (7.1%)	7 (9.1%)	14 (15.7%)	
Cold ischemia time (hours)	13.9						<0.001
0–8		7,328 (58.2%)	6,990 (58.3%)	201 (53.0)	60 (70.6%)	33 (61.1%)	
9–12		4,370 (34.7%)	4,159 (34.7%)	132 (34.8%)	21 (24.7%)	15 (27.8%)	
> 12		893 (7.1%)	833 (7.0%)	46 (12.1%)	4 (4.7%)	6 (11.1%)	

*HCC, hepatocellular carcinoma; CCC, cholangiocellular carcinoma; NET, neuroendocrine tumor; LSAR, sarcoma of the liver; SD, standard deviation; CNI, calcineurin inhibitor*.

Univariate analysis was performed using the Kaplan-Meier estimator. The Mantel-Cox log-rank test was used for comparisons between the groups. Kaplan-Meier curves for patient and graft survival including risk tables are available as supplements. Multivariable Cox regression analysis was conducted to account for the possible influence of the following confounders on the 5-year graft survival results: transplant year, recipient age, sex and race, geographical origin, donor age, antihypertensive therapy (i.e., no vs. yes), treatment for diabetes, history of smoking, immunosuppressive medication [i.e., the type of calcineurin inhibitors (CNI)—cyclosporine vs. tacrolimus, type of antiproliferative—azathioprine vs. mycophenolic acid, steroids—yes vs. no], cold ischemia time, marginal donor, urgency, general evaluation, type of donor death, number of diseases leading to transplantation (1 or 2) and graft size. Hazard ratio (HR) values with a 95% CI were calculated for the overall graft loss and mortality rates during the first five post-transplant years.

Comparisons between the demographics of analyzed groups were made using the Kruskal-Wallis rank-sum test for continuous and the chi-square test for categorized variables (Table 1). The software R 4.0.2 (R Development Core Team, Vienna, Austria) was used for statistical analysis. *P* < 0.05 were considered statistically significant.

## Results

Our analysis included 14,623 patients from 125 transplant centers in 27 countries. Of the 14,623 patients, 13,862 (94.8%) were categorized as HCC, 498 (3.4%) as CCC, 100 (0.7%) as NET, and 163 (1.1%) as LSAR. During the same period, 66,656 liver transplantations were performed due to other diseases so that cases in which rare malignant diseases led to liver transplantation accounted for 0.9% of all transplantations and 5.2% of the transplantations per our study parameters. The mean follow-up duration for all patients was 4.6 years.

### Graft and Patient Survival

**Figure 1:**
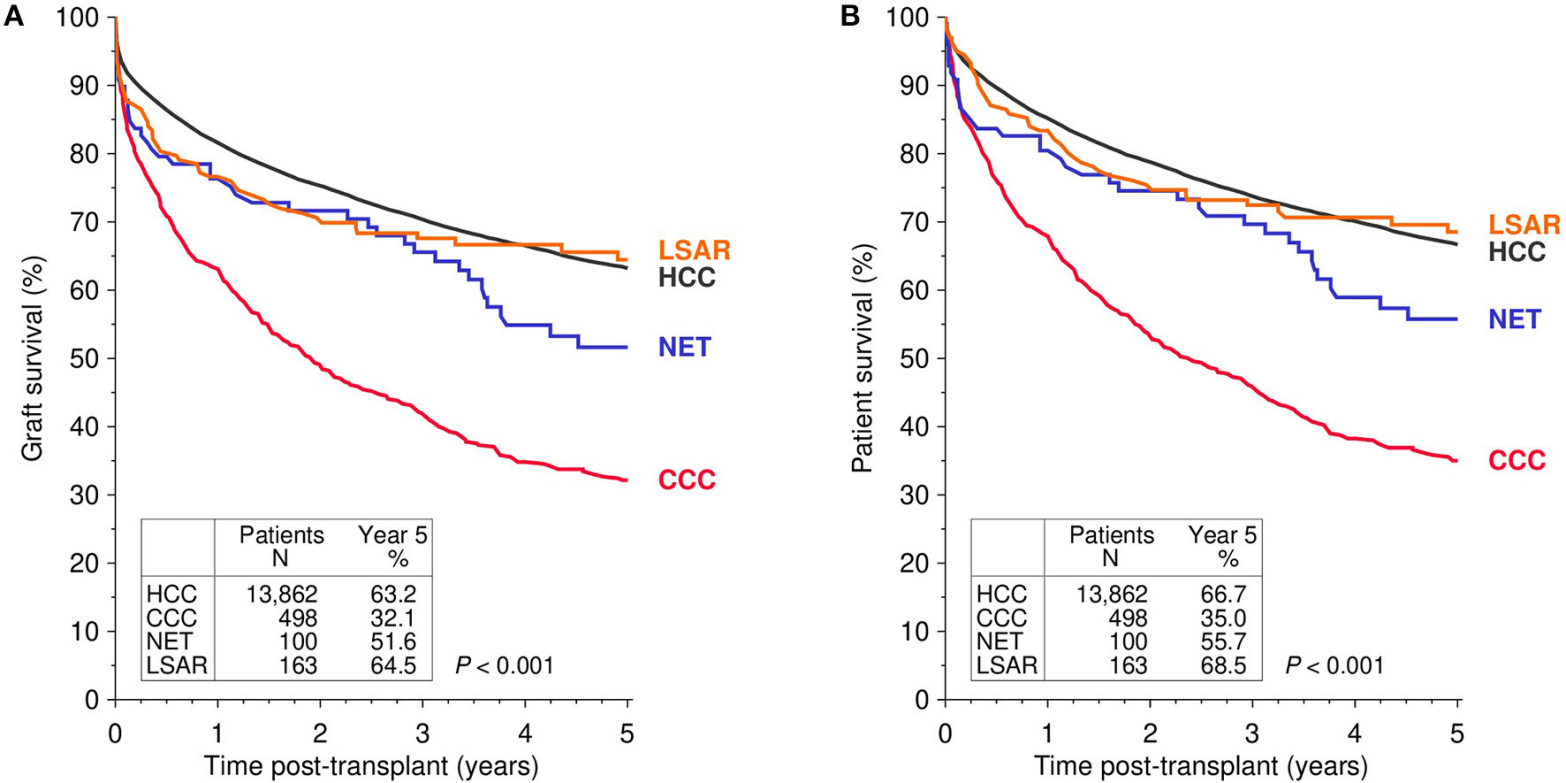
Kaplan-Meier analysis of graft **(A)** and patient survival **(B)** in first deceased donor liver transplants performed in patients with different pre-transplant malignancies that led to transplantation. The global log-rank *P-value* is shown. HCC, hepatocellular carcinoma; CCC, cholangiocellular carcinoma; NET, neuroendocrine tumor; LSAR, sarcomas of the liver.

Figure 1 illustrates the univariate Kaplan-Meier analysis of 5-year graft and patient survival in patients with pre-transplant malignancies that led to liver transplantation. Rarely occurring malignant diseases were compared to the more common malignancy HCC. Patients with LSAR seemed to have a relatively good and similar graft survival compared to patients with HCC, whereas patients with NET and particularly CCC exhibited diminished graft survival rates during the first five years post-transplant. Multivariable analysis confirmed the univariate findings (Table 2). As compared to the reference population of HCC patients, CCC exhibited a significantly higher risk of graft loss (HR = 1.77; CI 1.56–2; *P* < 0.001), whereas a slightly, but statistically not significantly increased risk was observed in patients with NET and LSAR (HR = 1.2; CI 0.88–1.65; *P* = 0.25, HR = 1.12; CI 0.84–1.48; *P* = 0.44, respectively). Similar results were obtained when patient survival was analyzed. Compared to HCC recipients, the risk of death was significantly increased in CCC recipients (HR = 1.88; CI 1.63–2.09; *P* < 0.001) and was insignificantly higher in NET (HR = 1.27; CI 0.92–1.77; *P* = 0.15) and LSAR recipients (HR = 1.11; CI 0.83–1.5; *P* = 0.48, Table 2).

**Table 2:** Results of multivariable Cox regression analyses for 5-year graft loss, patient death, and death due to cancer in patients with different pre-transplant malignancies that led to transplantation.

**Malignancy type**	**HR**	**95% CI**	***P***
Graft loss			
HCC	1 (Ref)	–	–
CCC	1.77	1.56–2.00	< 0.001
NET	1.20	0.88–1.65	0.25
LSAR	1.12	0.84–1.48	0.44
Patient death			
HCC	1 (Ref)	–	–
CCC	1.84	1.63–2.09	< 0.001
NET	1.27	0.92–1.77	0.15
LSAR	1.11	0.83–1.50	0.48
Patient death due to cancer			
HCC	1 (Ref)	–	–
CCC	2.56	2.09–3.13	< 0.001
NET	1.37	0.77–2.46	0.29
LSAR	1.30	0.81–2.07	0.28

*Recipients with hepatocellular carcinoma (HCC) served as reference (Ref). CCC, cholangiocellular carcinoma; NET, neuroendocrine tumor; LSAR, sarcomas of the liver; CI, confidence interval; HR, hazard ratio*.

### Death From Cancer

**Figure 2:**
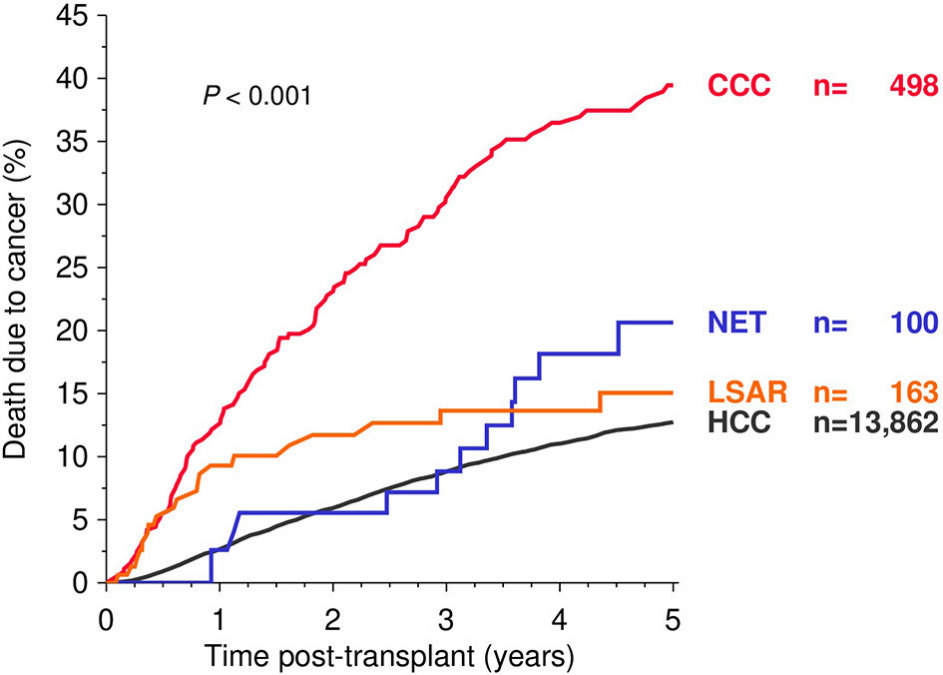
Kaplan-Meier analysis of death due to cancer during the first five post-transplant years. Global log-rank *P*-values are shown. HCC, hepatocellular carcinoma; CCC, cholangiocellular carcinoma; NET, neuroendocrine tumor; LSAR, sarcomas of the liver.

Compared to HCC recipients, a significantly higher rate of cancer-specific mortality was found in CCC as well as NET recipients in the Kaplan-Meier estimation (Figure 2). The multivariable analysis of death due to cancer, however, revealed a significantly higher risk only in recipients with CCC (HR 2.56; CI 2.09–3.13; *P* < 0.001), whereas NET and LSAR patients showed only a slightly increased risk (HR 1.37 and 1.30; CI 0.77–2.46 and 0.81–2.07; *P* = 0.29 and 0.28, respectively), without reaching the level of significance (Table 2).

## Discussion

Our findings confirm that HCC is, by far, the leading malignant indication in LT. Of the many cases analyzed over the 30-year period, only 761 cases, consisting of CCC, NET, and sarcoma altogether, from 125 transplant centers met the inclusion criteria for this study. The scarcity of these cases reflects the objections that many transplant physicians and surgeons have against malignancies other than HCC as an indication for LT. An excellent overview of this matter was conducted by the Innsbruck group (13). Here, the main concern regarding LT for intrahepatic CCC was, as mentioned above, that the excellent results of the Mayo protocol could not be reproduced broadly. The same accounts for LT in cases that did not fulfill the Milan NET criteria. Hemangiosarcoma of the liver has a disastrous prognosis after LT with a median survival of only 6 months and was, therefore, recently been proposed as an absolute contraindication for LT (14). Another report, in which the outcome of resection and transplantation in epithelioid hemangioendothelioma and hemangiosarcoma was compared, leading to the same conclusion, namely, that the prognosis in hemangiosarcoma is extremely poor and cannot be improved by LT (15). We observed unsatisfactorily low patient and graft survival rates for LT recipients with malignancies other than HCC. The minimal difference between the graft and patient survival rates in CCC and NET compared to HCC patients, deriving from very low re-transplantation rates and significantly higher rates of death due to cancer are indicative of tumor recurrence being the main limitation of survival in these patients.

It is remarkable that the 5-year graft and patient survival rates of CCC recipients in our study were as low as 32.1 and 35%, respectively. Generally, a 5-year graft survival rate of 50% is commonly considered acceptable for an indication for LT. In CCC, this benchmark was oftentimes only achieved in highly specialized institutions and in early-stage cases that had been subjected to standardized protocols, including neoadjuvant radiation and chemotherapy; here, the 5-year patient survival rate was 82% (9). In a recent retrospective multicenter study of small incidentally diagnosed CCC that did not undergo specific neoadjuvant protocols, the 5-year patient survival after LT was up to 65–69%, depending on the tumor size (16). The inferior graft and patient survival observed in our study in CCC recipients can have different reasons. Our finding that the risk of cancer-specific death was 2.56-fold substantially increased in CCC compared to HCC recipients suggests that tumor recurrence plays a significant role, even though exact information in this regard could not be retrieved from our study. Known risk factors for the dismal outcomes of CCC recipients in LT include tumor size >3 cm, multifocal growth in intrahepatic CCC, lymph node metastases, and the abandonment of the neoadjuvant radiation or chemotherapy, particularly in perihilar CCC cases (9, 17). These factors could not be evaluated because data on neoadjuvant protocols and localization of CCC are not available in CTS. More importantly, our study period started in 1988, whereas the Mayo protocol was initiated in 1994. When the first 10 years of our study period were abandoned and the 1998–2017 period was analyzed, the 5-year graft and patient survival rates improved from 32.1 to 40.2% and from 35 to 44.5%, respectively, remaining far below the rates observed at Mayo. Excellent, or even acceptable long-term survival rates after LT for CCC have so far only been reported in small series of highly selected patients undergoing aggressive neoadjuvant protocols. Larger, multicenter series reported unacceptably low 5-year survival rates of 23–47% (7, 18). The strict application of a neoadjuvant radio-chemotherapy protocol has the potential to improve the outcome in cases of perihilar CCC, yielding 5-year recurrence-free survival of 65% (19). A recent meta-analysis by Cambridge et al. confirmed this result with a 5-year survival rate of 65.1% after LT for perihilar CCC following neoadjuvant radio-chemotherapy (20). Therefore, the 2020 ILTS Transplant Oncology working group report recommends LT in cases of unresectable perihilar CCC in a standardized neoadjuvant protocol (21). The reason for the inferior outcome in our analysis remains uncertain. Omitting neoadjuvant therapy and the presence of advanced intrahepatic CCC led to significantly worse 5-year survival (20, 21). These conditions could not be analyzed in our data and could contribute to the observed differences. Interestingly, the graft half-life in our entire cohort of CCC recipients was 24 months still, which exceeded the known patient half-life under palliative therapy in non-resectable CCC by at least 12 months (22, 23). If there was an innovative chemotherapy protocol leading to survival results similar to those of LT in non-resectable CCC, it would be an outstanding improvement. Since the limitation in this context is the scarcity of liver grafts, the somewhat arbitrary threshold of 50% for 5-year graft survival exists for justification of LT, as other recipients could gain significantly longer, life-sustaining liver function with the same grafts (24).

The 5-year graft survival of 51.6% in NET patients in our study was substantially inferior to the 97.2% reported by Mazzaferro for LTs performed during 1995–2010. Therein, only stable liver metastases of previously resected primary G1/2 tumors, the metastatic volume of <50% of the liver, and preferably recipients <60 years of age were included in their specialized protocol for LT in NET (25). Unfortunately, since additional information on individual oncological circumstances, such as the site of the primary tumor, tumor stage and grading, and information about the medical treatment course of the NET, is not available in the CTS database, it remains unclear to what extent the recipients in our study fulfilled the Milan eligibility criteria for LT in hepatic, metastasized NET (26). The slightly higher age in our NET population is unlikely to account for the observed outcome differences. It is worth mentioning that there is also a discrepancy in the study periods; the Milan NET protocol was initiated in 1995, whereas our study period started in 1988. Individually looking at the different periods in our study, we observed an improvement of the 5-year graft survival rate in recipients with NET from 39.1% during 1988–1999 to 57.8% during 2000–2017. The results from the United Network for Organ Sharing (UNOS) also show an incline in 5-year survival after LT for NET metastases from 48% during 1988–2008 to 63% during 2002–2014 (27, 28). An analysis of the outcome of LT for NET from the European Liver Transplant Registry (ELTR) from 2013 revealed a 5-year patient survival rate of 52% (29). These results are in line with our 5-year overall survival of 55.7% through the entire study period.

The outcome of LT in LSAR varies widely depending on the histological subtype, with a good prognosis for hepatic epithelioid hemangioendothelioma (HEHE) and very limited post-transplant survival for hepatic hemangiosarcoma (15). Without more detailed information on the LSAR subtype, it is difficult to compare our results to previously published outcome data obtained for LT in LSAR. Risk factors for early tumor recurrence in HEHE, such as macrovascular invasion, LT within a 120-day or less waiting period, and hilar lymph node involvement, could not be retrieved from the CTS database (30). In our study, the 5-year graft survival in LSAR cases was comparable to that in HCC (LSAR vs. HCC; HR = 1.12; *P* = 0.44). The long-term graft survival of recipients categorized as LSAR in our study was comparable to the results of LT reported previously for HEHE (31). Based on the aforementioned, results for LSAR in our analysis have to be interpreted with utmost caution.

As the mammalian target of rapamycin (mTOR) inhibitor-based immunosuppression seems to show improved recurrence-free survival and better overall survival rates in HCC recipients of LT, their use in malignant indications is of great interest (32, 33). Given the high rate of missing values and immunosuppression stated as “other” without information when mTOR inhibitors were administered, we cannot elucidate the use of different immunosuppression protocols in our large cohort of LT recipients for malignant indications.

Some important conclusions can be drawn despite the above-mentioned limitations. Based on the composition of the centers participating in the CTS, our results are more likely to represent the clinical reality of liver transplantation outside the highly specialized high-volume centers (12). Interestingly, the estimated median patient survival of 28.5 months in CCC patients who received a liver transplant at centers with different grades of specialization still outperforms the 9.5–11.2-month patient survival achieved with the current palliative chemotherapy protocols with gemcitabine + platinum (22, 23). This could be pivotal in the decision-making process for transplant physicians in regions with little to no donor organ scarcity.

Our data also indicate that the general outcome of LT for NET might not reach the excellent patient survival rates of 97.2% at 5 years and 88.8% at 10 years that were reported from high-volume centers in transplantations performed during 1995–2010 (25). Indeed, the 5-year graft survival rate of 51.6% in our study was below the 60% rate reported for LT in non-resectable, yet stable hepatic metastases of colorectal cancer, which has been long abandoned as an indication for LT (34). With strict recipient selection, the Oslo group recently reported that 5-year survival rates of more than 80% can be achieved after LT for irresectable colorectal liver metastases (35). This challenges the exceptional status of metastasizing NET as the only indication for LT in secondary hepatic malignancy. If the outcome of LT for metastasizing NET for the majority of potential recipients is closer to the results reported herein than to the results from one of the few specialized high-volume centers, the option to remain on a palliative chemotherapy course could be a viable alternative for some patients, as it offers comparable long-term patient survival (60-month patient survival after LT in CTS: 55.7 vs. 45.9% and median patient survival of up to 84.7 months with palliative treatment) (36, 37).

In summary, LT offers an outcome comparable to that obtained via palliative treatment in NET and superior long-term survival relative to palliative chemotherapy in CCC. In light of organ scarcity, the role of HCC as the unique relevant malignant indication for LT seems justified, as cancer-specific mortality rates for LSAR, NET, and especially CCC, are significantly worse.

## Data Availability Statement

The datasets presented in this article are not readily available because the analyzed information are integral datasets within the Collaborative Transplant Study. Requests to access the datasets should be directed to philipp.houben@ukmuenster.de.

## Author Contributions

PH, SS, and CS participated in research design, data analysis, and writing of the paper. CU participated in the performance of research, data analysis, and writing of the paper. BD participated in data analysis and performance of research. AM participated in data analysis. All authors contributed to the article and approved the submitted version.

## Conflict of Interest

The authors declare that the research was conducted in the absence of any commercial or financial relationships that could be construed as a potential conflict of interest.

## Publisher's Note

All claims expressed in this article are solely those of the authors and do not necessarily represent those of their affiliated organizations, or those of the publisher, the editors and the reviewers. Any product that may be evaluated in this article, or claim that may be made by its manufacturer, is not guaranteed or endorsed by the publisher.
