# Supplementary figures and images for: Rare Malignant Indications for Liver Transplantation: A Collaborative Transplant Study Report
Source: Front Surg. 2021 Dec 3;8:678392. doi: 10.3389/fsurg.2021.678392 (PMC8678034; doi:10.3389/fsurg.2021.678392)

(A)

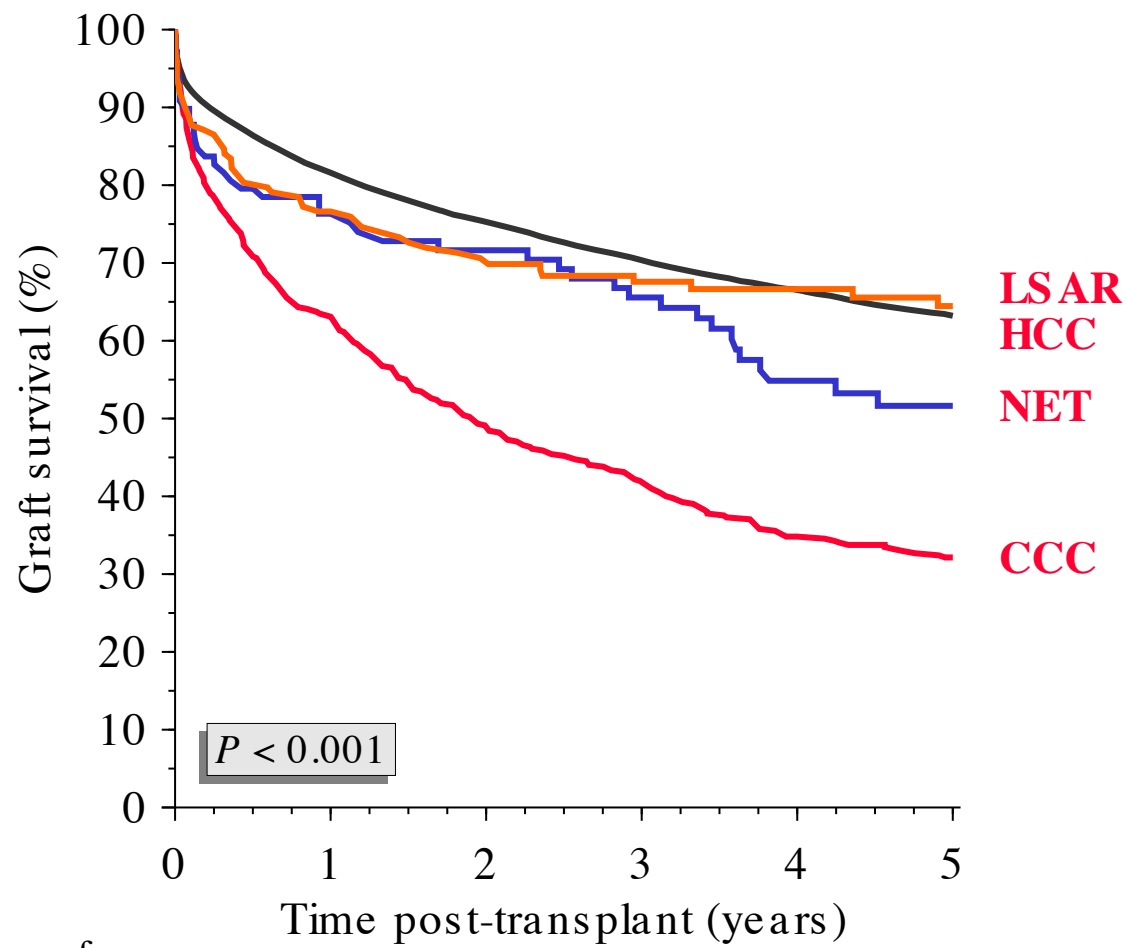

Number of  
transplants

|      |        |        |       |       |       |       |
|------|--------|--------|-------|-------|-------|-------|
| HCC  | 13,862 | 10,816 | 9,160 | 7,706 | 6,539 | 5,482 |
| CCC  | 498    | 310    | 225   | 181   | 141   | 120   |
| NET  | 100    | 71     | 61    | 54    | 41    | 32    |
| LSAR | 163    | 124    | 106   | 89    | 71    | 59    |

Supplement: Supplementary file 1 [file Data_Sheet_1.PDF]

(B)

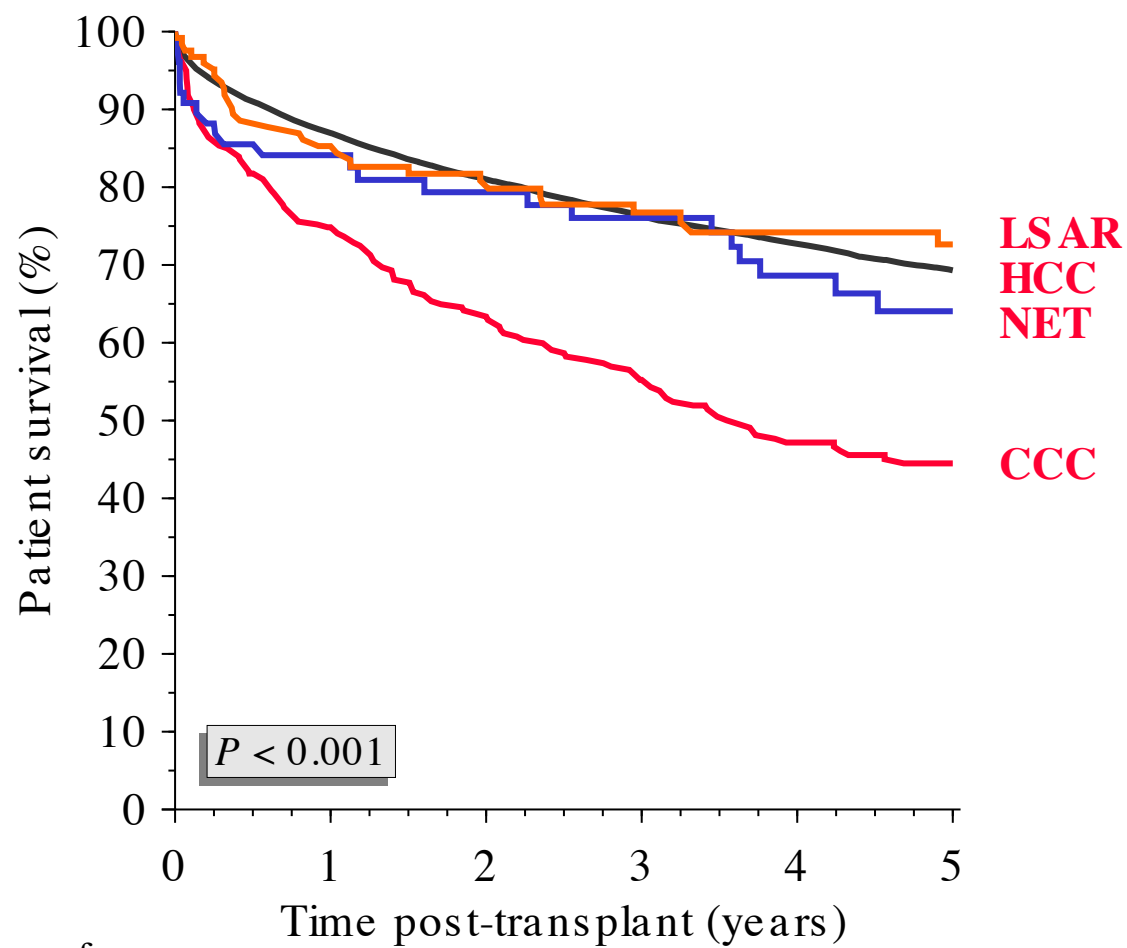

Number of  
transplants

|      |        |        |       |       |       |       |
|------|--------|--------|-------|-------|-------|-------|
| HCC  | 12,297 | 10,163 | 8,628 | 7,243 | 6,135 | 5,097 |
| CCC  | 281    | 206    | 160   | 129   | 99    | 83    |
| NET  | 78     | 60     | 50    | 46    | 37    | 27    |
| LSAR | 123    | 104    | 91    | 75    | 58    | 47    |

Supplement: Supplementary file 2 [file Data_Sheet_2.PDF]

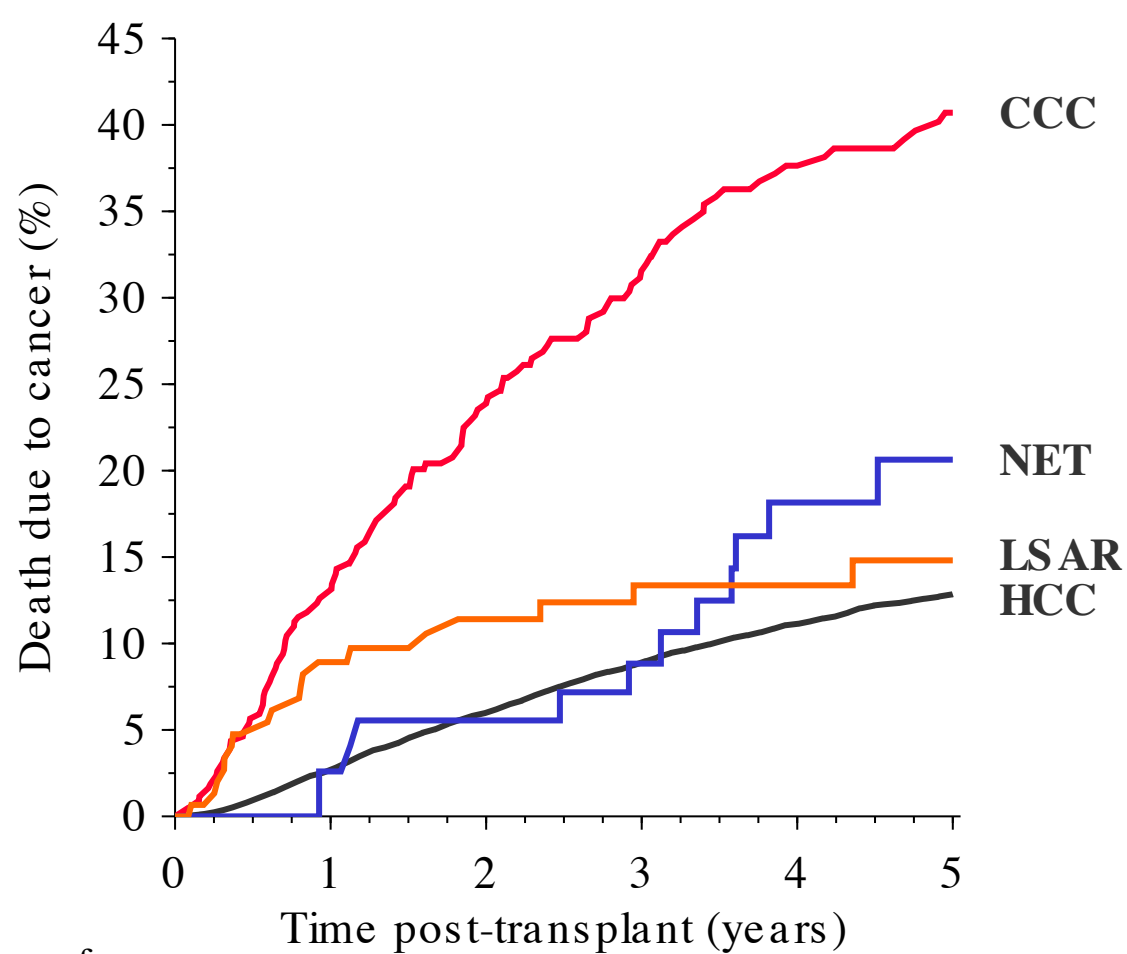

Number of  
transplants

|      |        |        |       |       |       |       |
|------|--------|--------|-------|-------|-------|-------|
| HCC  | 13,651 | 11,143 | 9,122 | 7,675 | 6,519 | 5,460 |
| CCC  | 478    | 322    | 218   | 173   | 136   | 114   |
| NET  | 100    | 75     | 61    | 55    | 41    | 32    |
| LSAR | 157    | 131    | 105   | 88    | 70    | 58    |

Supplement: Supplementary file 3 [file Data_Sheet_3.PDF]
